# OSCANN: Technical Characterization of a Novel Gaze Tracking Analyzer

**DOI:** 10.3390/s18020522

**Published:** 2018-02-09

**Authors:** Erik Hernández, Santiago Hernández, David Molina, Rafael Acebrón, Cecilia E. García Cena

**Affiliations:** 1 Aura Innovative Robotics, 28045 Madrid, Spain; ehernandez@aurarobotix.com (E.H.); santiago@aurarobotix.com (S.H.); dmolina@aurarobotix.com (D.M.); rafael@aurarobotix.com (R.A.); 2 Centre for Robotics and Automation, UPM-CSIC, José Gutiérrez Abascal Street, 28006 Madrid, Spain

**Keywords:** eye-tracker, neurodegenerative diseases, medical sensor, video electro-oculography, oculomotor movements, clinical practice, early diagnosis

## Abstract

Eye-movement analysis has grown exponentially in recent decades. The reason is that abnormalities in oculomotor movements are usually symptoms of injuries in the nervous system. This paper presents a novel regulated solution named OSCANN. OSCANN aims at providing an innovative tool for the control, management and visualization of oculomotor neurological examinations. This solution utilizes an eye-tracker sensor based on video electro-oculography (VOG) technology to capture eye movements and store them in video files. Such a sensor can store images at a rate of 100 frames per second. A characterization study was performed using twenty-two volunteers (13 male, 9 female, ages 22–45 years, mean 29.3 years, SD = 6.7) to assess the accuracy and precision specifications of OSCANN during oculomotor movement analysis. The accuracy was evaluated based on the offset, whereas precision was estimated with Root Means Square (RMS). Such a study reported values lower than 0.4${}^{\circ }$ and 0.03${}^{\circ }$ of accuracy and precision, respectively. These results suggest that OSCANN can be considered as a powerful tool to measure oculomotor movement alterations involved in some neurological disease progression.

## 1. Introduction

Eye-movement analysis has grown exponentially in recent decades. As a result, a great deal of devices have been developed for research purpose in psychology and neurology fields. Such interest relies on the fact that the correct functioning of oculomotor movements in human beings depends on both psychological and neurological factors. Firstly, it explains the ability to perceive and interpret the environment. Secondly, it demonstrates the capability to perform specific actions according to a plan. Thirdly, it comprises an accurate control system for the correct execution of the orders transmitted.

Several neural systems interact among them to control the suitable performance of the ocular and ocular-cephalic movements. For example, brain regions such as cortical networks, basal ganglia, brainstem nuclei and the vestibular system are organized to produce different eye movements. Such movements aim, for example, to maintain the position of maximum visual acuity on objects of interest. The proper execution of this task is essential for the daily life of human beings. This control system has an extremely complex behavior. As a consequence, the appearance of abnormalities in oculomotor movements is expected due to injuries in the nervous system.

Abnormalities in oculomotor control are associated to many neurodegenerative diseases. Therefore, eye-movement examination could be valuable to assess an accurate diagnosis of these diseases. In addition, an objective and structured examination can help to generate an earlier diagnosis. Even more importantly, valuable information about their progress can be obtained by performing this task periodically [1]. In order to properly measure eye movement, it is necessary to separate the pure ocular movements (OMs) from those combined with head movements widely known as ocular-cephalic movements (OCMs). At present, the most sophisticated sensors used to measure eye movements implement mechanisms to fix the head. The objective of such fixation is to achieve high precision measurements.

It is widely recognized that the gold standard in eye-tracker sensors is the so-called magnetic coil system, which was developed during the 1960s [2]. The nature of this sensor is invasive due to the use of a copper wired contact lens placed around the pupil. That system captures the voltage generated by the lens during its movement inside an external magnetic field generated by another coil. The lens has three different sets of coils for generating signals in horizontal and vertical directions as well as the torsional movement of the pupil. That system also needs to fix the head so that the movements recorded from the coils are produced exclusively by the rotations of the eye. In order to create an innovative and less invasive measurement system, other technologies have been developed. Included among these technologies are electro-oculography (EOG) and video electro-oculography (VOG).

In [3], an eye-tracker sensor based on electro-oculography (EOG) is presented. Such a sensor needs at least two pairs of electrodes to measure horizontal and vertical eye movements. Moreover, an extra electrode is placed on the forehead of the patient which serves as a reference signal. One of the main advantages of EOG systems is the high sample rates of the biological signal. However, the absolute value of this signal is low. In order to mitigate this problem, amplifiers and filters must be applied. As a consequence, accuracy is poor. In addition, EOG systems are also considered as invasive because electrodes must be placed on the skin of the patient.

On the other hand, in video electro-oculography (VOG), at least one camera is used to capture the eye movements. VOG systems are widely used because they are non-invasive and provide high accuracy measures of oculomotor movements. Devices based on the VOG principle can be divided into two types: fixed or wearable [4].The former use a camera mounted outside the patient body, whereas the latter attach a camera to the human body by means of, for example, glasses or a helmet.

As observed, technological developments allow these types of sensors to be improved to make them non-invasive, more comfortable and accurate. However, recent studies address the price as the biggest issue of most of those sensors. Certainly, the price of these devices must be low in order to be used in clinical practice. Nonetheless, most commercial eye-trackers are still very expensive. In addition, the human–machine interfaces used by those systems are not friendly at all [5,6]. For this reason, Hansen and Ji [7] conclude that research remains the only application field for these sensors.

This article describes a novel gaze-tracker device named OSCANN, along with the methodology utilized to obtain its technical characterization. OSCANN can be classified as a VOG system that records the oculomotor movements at 100 frames per second. It is worth mentioning that the device and method are patent pending (PCT/EP2017/060776). At present, OSCANN is being used in six hospitals in Spain and the device fulfils all the legal requirements according to European regulation for medical devices. These clinical trials include pathologies such as Alzheimer’s, Parkinson’s, cirrhosis, diabetes type II, minimal hepatic encephalopathy and epilepsy [8,9,10].

It is important to note that the main difference between OSCANN and other similar systems relies on the hardware and software design. That is because both of them were developed to be used by non-experts in science or technology. Therefore, OSCANN aims to become a powerful device for the early diagnosis and monitoring of neurodegenerative disease in clinical practice. In other words, OSCANN is not only a sole eye-tracker but also a complete regulated solution aimed at providing assistance to control, visualize and manage oculomotor neurological examinations. In addition, this sensor can be easily installed in a clinical environment. Moreover, due to its IR illumination system, the sensor remains immune to natural or artificial light. Finally, OSCANN software has been developed to meet the needs of neuroscientists. As a result, an broad set of visual tests has been created, each aimed at generating different oculomotor responses. Also, they are simple enough for patients to understand them easily.

Section 2 of this work describes the materials and methods utilized to perform the characterization study of OSCANN. Section 3 describes the experimental setup. Section 4 presents the evaluation and experimental results. Section 5 concludes this work.

## 2. Materials and Methods

**Figure 1:**
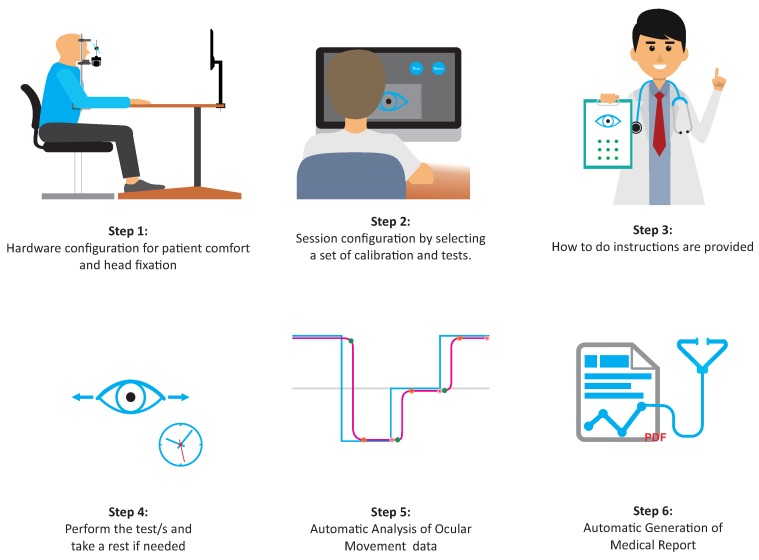
Overview of the steps required for optimum use of OSCANN.

The study of eye movements must be performed using specific neuropsychological tests. Generally speaking, during these tests, a stimulation scene must be presented to the patient while his/her oculomotor response is registered. OSCANN is fitted with all the necessary components to conduct this task. The description and functionality of such components are described below. Figure 1 shows an overview of the steps needed to obtain successful results with OSCANN. This overview is used throughout this section to illustrate the description provided. Such a description is divided into two parts, namely hardware and software. From now on, the person in charge of OSCANN will be called indistinctly medical personnel, operator or instructor. The person performing neurological tests will be denoted as patient, volunteer or participant.

### 2.1. Apparatus

In order to perform a test using OSCANN, a patient must be sited in front of the so-called stimuli monitor (Step 1 in Figure 1). Such a monitor must be placed at a fixed distance of 60 cm with respect to the patient examined eye. Using this configuration, the patient is able to watch the stimulus scene whereas her/his oculomotor movements are recorded in raw data video format. Such a recording is then used to perform a detailed data analysis. Thus, for example, subtle artifacts such as eye blinks can be detected.

**Figure 2:**
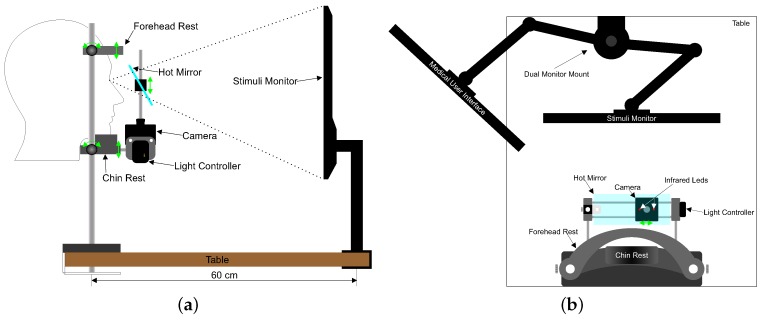
OSCANN Desk 100. (**a**) Side View; (**b**) Top View.

OSCANN composition is detailed in Figure 2. A chin and forehead rest are used to immobilize the head of the patient. In addition, a belt is used to keep the head fixed to the chin rest. The use of these elements relies on their ability to minimize head motion which, in turn, ensures accurate data acquisition. Moreover, the forehead and chin rest can be adjusted for adaptability to different head anatomies. Such adaptability aims at providing maximum patients comfort. As was suggested in [11,12], forehead and chin rest are widely considered key components to stabilize the head of patients.

External conditions such as artificial light can considerably decrease the resolution of images. For this reason, near-infrared (NIR) illumination is used while recording the eye. In spite of this, room lighting as well as noise and temperature should be homogeneous to avoid distracting the patient. The illumination system of OSCANN is based on two light-emitting diodes installed inside the camera compartment. These diodes are used not only for the purpose of lighting but also for gaze estimation. Corneal reflection, usually called glints, is widely used to correct slight head movements [13]. Such glints must be inside the pupil or iris area so that they can be detected. For estimation purposes, the average of their position reduces error. It is worth noting that light intensity is controlled by software using a light controller. It is also important to note that such intensity is at maximum 100 mA. This intensity is limited to ensure compliance with the photo-biological safety condition, UNE-EN 62471. Furthermore, the illumination system complies with the values of radiance and irradiation required to be considered as a risk-free system according to IEC 62471-2.

As shown in Figure 2, a hot mirror is located in front of the patient eyes at 45${}^{\circ }$ with respect to the chin rest. The use of this component allows images to be captured as if the camera were located directly in front of the examined eye but leaving the visual field free of obstacles. The reason is that the NIR light emitted by the illumination sources aforementioned is reflected by the hot mirror and captured by the camera. The position and orientation of the camera perpendicular to the visual axis of the patient depends mainly on such reflection.

Additionally, OSCANN uses a high-speed infrared USB 3.0 camera to record oculomotor movements. Such a camera is placed on a rail which allows horizontal displacement to focus on the dominant right or left eye. Furthermore, in order to improve precision, the camera was installed as close as possible to the eye position. It is worth mentioning that the camera as well as the hot mirror move vertically along with the chin rest support. Finally, the whole structure of this medical sensor must be attached to a standard desk with enough space for distribution specifications.

The design of Oscann specifies the use of an additional monitor which is defined as Medical User Interface (Figure 2b). Such a requirement relies on the task itself, i.e., the presentation of a visual stimulation scene to the patient. This stimulation must not only be presented to the patient but must also be configured previously by the operator of the equipment. Therefore, in order to avoid disturbance for the patient, both actions (namely, display and configuration) are performed separately.

### 2.2. Software

**Figure 3:**
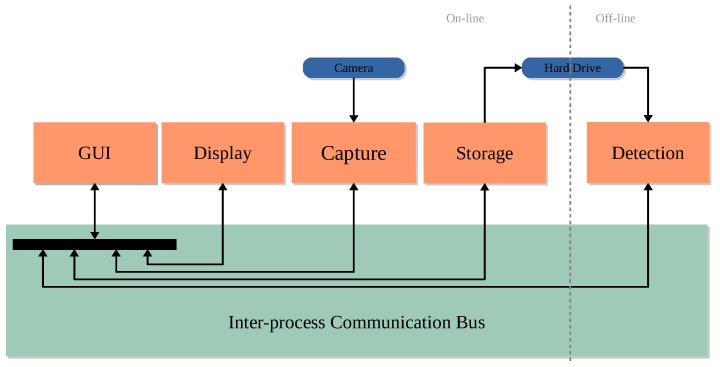
Interoperability and the architecture scheme of the software components to handle the hardware of OSCANN.

According to what Bedell and Stevenson [9] state: “The manufacturers of these instruments should be encouraged to provide appropriate interfaces to allow for eye-movement signals to be sampled, stored, and visualized so that this additional functional capability can be used to the best clinical advantage”, Oscann provides a software suite with a set of features that fulfil such requirements. The architecture of such a suite is shown in Figure 3. As can be observed, this architecture comprises multiple computer programs or processes running on the same machine (rectangles with dashed lines). Moreover, these processes use a logical bus to establish required communication channels to exchange information and control actions. Before describing the objective of each process, it is important to list the actions that can be performed by using OSCANN Suite.

The operator can select as many tests as necessary (Step 3 in Figure 1). Generally speaking, depending on the test, a specific stimulus scene is presented to the patient. In this light, it is important to note that OSCANN suite has the following neurological tests available: visually-guided saccades, visually-guided anti-saccades, visually-guided memory saccades, visually-guided memory anti-saccades, countermanding, fixation, fixed image, pupillary light reflex, smooth pursuit and optokinetic. All these tests can be displayed vertically and horizontally. Moreover, smooth pursuit has special configurations such as sinusoidal, oblique and circular. Finally, optokinetic has two presentation forms, namely top-down and right-left. Beyond the direction, all these tests have some specific features that can be customized. For example, the time of saccade in a saccade-based test, regardless of its type: pro, anti or memory. In fixation, the test duration in seconds. In fixed image test, the duration in seconds and the image to be presented. In pupillary light reflex, the time between black and white changes. Finally, in smooth pursuit, the animation speed in hertz.

On the other hand, in order to keep high levels of accuracy, it is mandatory to perform a calibration at least once every three test. However, a maximum of three tests can be conducted per calibration, as long as the patient head remains still. Generally speaking, a calibration establishes a mapping or a mathematical relation between the features detected in the eye image and the position of gaze in the stimulus space [14]. In other words, calibration data is necessary to show the results of each test. In a calibration, which could also be used as a test, the patient must direct her/his gaze to the stimulus presented in the stimuli monitor during a short period of time. The positions of such a stimulus form a grid that covers the whole monitor. At each position, the color of the stimulus changes from red to green. Red color is used to capture the patient attention, whereas green color indicates the beginning time of the data acquisition. On the other hand, the data captured at each position is used to calculate the coefficients of a bi-quadratic non-linear mapping function by means of a least-squares method. That is, OSCANN implements a regression-based approach for gaze estimation. Moreover, OSCANN Suite provides two types of calibrations, namely automatic, and combined. These options provide more or less control to the operator over the calibration. Such a level of control over calibration aims at improving quality in data acquisition [15]. In an automatic calibration, the operator only indicates the beginning of the calibration process. After that, the stimulus changes automatically in position and color from red to green. On the other hand, in a combined calibration, the stimulus also changes automatically in position but the operator decides at each position when the data acquisition begins.

Once everything has been properly configured, the testing procedure begins by instructing the patient how to behave during both calibration and consecutive tests (Step 3 in Figure 1). Once such a procedure starts, eye movements are captured and stored in a video format file for further processing, viewing and analysis (Step 4 in Figure 1). File saving settings are decided by the operator. All of these actions, procedure configuration, stimulus display, eye movements capture and video storage are executed on-line (white area in Figure 3). Time per examination relies on the number of tests conducted. Further on, the operator needs to process the information captured, which unlike previous steps, is carried out in an off-line setting (gray area in Figure 3). It is important to note that such a task is performed off-line and is denoted with a gray area in Figure 3.

OSCANN Suite offers a wide range of tools to view processed data. For example, this data is presented graphically by means of, depending on the test type, line, scatter or color plots. An important feature of data viewing is that it allows to the oculomotor movements captured by means of video preview to be replayed. This task can be performed in different manners including play modes or mouse interaction over graphs by clicking or dragging and specific areas. As stated by Enright [16]: “replay of a videotape permits repeated visual examination of actual performance, so that subtle artifacts can be detected (e.g., miniature eye blinks that can deflect the eye but do not reach the pupil or lateral margins of the cornea)”.

Automatic data analysis features are also included in OSCANN Suite (Step 5 in Figure 1). The characteristic values extracted by such analysis depend, once again, on the test type. For example, in a visually guided saccades test, parameters such as latency, gain and maximum peak are calculated. This information is computed both individually, saccade by saccade, or statistically over the whole test. It is important to mention that this analysis recognizes and disables parts of the test in which the task has not been performed properly. On the other hand, anti-saccades tests analysis provides statistical data about correct and incorrect anti-saccades as well as reflexive saccades along with latency, gain and so forth. Note that each test has special features to be extracted by the analysis module. OSCANN Suite includes the algorithmic processes to obtain all these features. Such automatic processes are mainly based on the velocity principle but dispersion is also used to extract features of a fixed image test. Finally, all this information is used to generate a medical report in pdf format (Step 6 in Figure 1).

It is important to highlight that an operator can configure and control as many test procedures as needed by means of the GUI module of OSCANN Suite. This module is also useful to control the near-infrarred light (NIR) source as well as previewing the captured image to ensure a correct image source acquisition. Additionally, this module allows to process, view, and analyze the data. Furthermore, it internally controls the capturing and storing processes. This is the reason why it has a bidirectional communication with the other processes. The control flow implemented to perform, for example, a test, is as follows: Firstly, GUI module indicates to the stimuli module that a stimulus must be displayed. Secondly, at the same time, depending on the stimulus state, it is indicated to the capture module (or not) that images must be acquired. Finally, the capture module sends images to the writer module to be stored on the disk. In other words, the GUI module is the core of OSCANN Suite.

Finally, some PC specifications are required to achieve optimal functioning of OSCANN: Core i7 Intel processor or equivalent, 8 GB of RAM and native USB3.0 controllers to connect the camera and light modules.

## 3. Experimental Setup

Sampling rate and data quality are the most important technical specifications of eye-trackers. In turn, the latter is influenced by precision and accuracy, which is also called offset. In eye-tracker terminology, precision represents the consistency of consecutive estimated position of gaze points, whilst accuracy refers to the difference between true and estimated gaze positions. Precision and accuracy have a direct impact on data quality. The higher the precision and accuracy, the higher the data quality. Moreover, high data quality is required for the improvement of some features and the performance of some specific tasks. For example, low precision could affect eye velocity calculations. This fact makes more it difficult to distinguish dynamic eye movements such as small saccades from fixations. Furthermore, accuracy has an impact on uncertainty, which involves problems associated with imperfect information. As might be expected, technical specifications of eye-trackers determine the field in which these types of equipment can be used. In this vein, Nyström et al. [15] state that “in clinical applications, high precision is critical to investigate imperfections in the oculomotor system”. Additionally, those authors claim that high accuracy is essential for neurological research studies.

Sampling rate specification relies on the optical device used for capturing images. However, precision as well as accuracy must be validated through a complete characterization study. Such a study is required to validate the technical specifications of the medical equipment described previously, namely OSCANN. This validation process is very important due to the task performed. As aforementioned, such a task involves identifying cognitive symptoms of dementia, which requires high data quality. This section presents the methodology implemented to perform such a characterization study. In this methodology, all variables such as operators, participants, and experimental setup have been taken into account. The characteristics of all these factors are described below. Then, a detailed description of the procedure implemented is provided.

### 3.1. Operators

Operators are key to the optimal functioning of any medical device including OSCANN. They perform several tasks and make critical decisions during the data acquisition process. Firstly, they must configure the medical device for adaptation to the physiognomy of each patient. Secondly, they must decide whether the image quality is suitable or not. In the case of low quality, they must focus the eye camera in order to obtain high quality eye images. Thirdly, they determine whether to accept, cancel or repeat a calibration. It is worth noting that operators have real -time feedback about how a calibration has been performed in order to make this decision. Fourthly, they must provide instructions to the participants about how to behave during calibrations and tests. In this light, in order to avoid differences in eye-movement behavior, the instructions provided to each participant must be exactly the same. This is mainly because instructions directly affect the top-down control of eye movements [17]. Finally, operators must handle the whole process deciding the initial and final time of each calibration or test as well as the resting time for the participant. The whole characterization study of OSCANN was performed by a highly qualified operator (data set A) and by a novice (data set B) who followed the instructions provided in the user manual of OSCANN. It is widely accepted that experienced operators generate data with better overall quality than novices [15].

### 3.2. Participants

Twenty-two volunteers took part in the characterization study of OSCANN (13 male, 9 female, ages 22–45 years, mean 29.3 years, SD = 6.7) All volunteers were inexperienced in eye movement research and eye tracking use. All of them cooperated by performing the required tasks as well as possible. Moreover, such cooperation implied that volunteers were not allowed to use make-up. It is worth noting that, most of the time, pupil detection can be performed correctly using this type of products. However, their use can sometimes cause problems. For example, make-up makes some important eye features difficult to detect. On the other hand, glasses produce reflections as well as occlusions which also made tracking eye movements more difficult. All volunteers had both normal (or corrected-to-normal) vision acuity and color vision. Only five of the volunteers used contact lenses. Volunteers participated in sessions that lasted approximately ten minutes. Informed consent was provided by each volunteer after an explanation of the nature of the study.

### 3.3. Stimuli

Stimuli, also called targets, were bright green circles with a total outer diameter of 0.5${}^{\circ }$ of visual angle. Each stimulus was designed with a small white center cross on which to focus. They were of high contrast to enhance their perception. Stimuli were displayed individually for 1500 milliseconds on a black background. To this end, a conventional screen display model Benq GW2270 was used.

### 3.4. Artificial Eye

Oculo-motor movements in human beings include intrinsic noise. This is mainly because the eye is never completely stationary [18]. An approach widely used in literature to measure free-of-noise precision consists in using artificial eyes [19]. The main advantage of this approach is the deduction of noise produced by microsaccades, eye drifts or tremor. However, precision analysis using only artificial eyes can be misleading. The reason is that artificial eyes do not reflect the diversity and physical properties of human-being eyes [20]. The use of artificial eyes can therefore be considered as complementary. Thus, in order to enhance the characterization study of OSCANN, a simple artificial eye was used. It comprises of a high resolution flat 2D image printed on a paper which represents the iris, pupil and corneal reflexions. Such an image was obtained using a sample image of the eye captured with OSCANN. The printed image was attached to OSCANN using a rigid support. This support allows the artificial eye to be positioned in a location similar to that of a human-being eye. Thus, the recorded images using this artificial eye were captured through the hot mirror. It is worth noting that the pupil detection algorithm of OSCANN could perfectly detect this special artificial eye.

### 3.5. Environment

Illumination in the test environment is very important to obtain adequate accuracy. In fact, some eye trackers suggest the use of external light sources such as a soft box [21] or active IR [22] illumination to mitigate reflexion problems or enhance lighting conditions. It is worth noting that OSCANN does not need any external device related with illumination. That is, OSCANN can be used under standard working conditions. In order to validate this assumption, the characterization study of OSCANN was carried out in an ordinary office room. Such a room was illuminated with a standard widely used fluorescent light. It is important to mention that this light source does not produce enough infrared light to affect eye recordings [23]. To minimize distraction and increase patients’ comfortability, OSCANN was installed in an independent room where only the operator and patient were allowed to be during the examination.

### 3.6. Procedure

This characterization study was strictly performed according to the procedure shown in Figure 1 and described previously. Firstly, the operator asked each participant to sit in front of OSCANN, followed by a simple configuration for adaptability and comfort purposes. When it was needed, the operator also adjusted the position of the hot mirror and the focus of the camera to improve both pupil detection and corneal reflection. Such detection was verified by asking the volunteer to gaze at the corners of the stimuli monitor. Accurate detection in the corners is necessary to obtain high quality data during the analysis.

Secondly, a nine-point calibration, an eighteen-point calibration-like test and a fixation test were selected and configured by the operator. For both, calibration and calibration-like tests, stimuli were displayed for two seconds each. However, in order to avoid erroneous anticipated saccadic movements, data was captured 500 milliseconds after stimulus onset. The fixation test was configured to last fifteen seconds. The eighteen-point calibration-like test was used in this characterization study because it allows the extraction of fixation points that uniformly cover the entire stimuli monitor. Stimuli for this test were randomly shown in order to minimize the number of anticipatory saccades. On the other hand, the fixation test consisted of a single stationary stimulus centered on the screen.

Thirdly, the operator instructed each volunteer how to behave during the calibration and each test. Standardized instructions were used. For the calibration and calibration-like test: “Look at the stimulus; as soon as a new stimulus appears, look at it as fast as you can until it disappears. Do not anticipate to a new location on the display”. For the fixation test: “Look at the stimulus until it automatically disappears”. Instructions were enhanced by pointing at the stimuli screen to emphasize the target positions and the correct responses.

Since a high quality calibration is mandatory, the operator visually verified the gaze positions with regard to the stimuli screen to decide whether or not the calibration must be accepted or repeated. This step is crucial because poor quality calibration will result in poor quality data.

### 3.7. Data Analysis

Some undesirable situations that affect the quality of the data may occur during the data acquisition process. Blinks are examples of these types of situations. The samples related to these situations must be excluded from the data set. In this vein, all the images captured that coincided with the exclusion criteria were previously removed from the data set. Pupil occlusion arises at the beginning and end of a blinking event. On average, pupil occlusions lasted approximately two data samples. Additionally, those samples where the gaze was more than 3${}^{\circ }$ away from its target were also excluded from the analysis. Finally, five samples after stimulus onset were excluded from the data set in the calibration-like test.

At the end, only 7777 samples were excluded out of a total of 92,674 samples of the data set. To be precise, 6827 samples from the calibration-like tests and 950 samples from the fixation tests were discarded which represent 11.57% and 2.81% of the total, respectively. Nearly 91% of the samples recorded were listed as high quality. In other words, 91% of the analysis was successful.

### 3.8. Accuracy

Accuracy was calculated based on the average angular offset of the eighteen-point calibration-like tests. Thus, given *n* intended fixation targets, accuracy can be obtained from

(1) $$\theta_{Offset}=\frac{1}{n}\sum_{i=1}^{n}\theta_{i}$$

where $\theta_{i}$ is the angular distance between *m* calculated fixation locations and the location of the corresponding intended fixation target and is given by

(2) $$\theta_{i}=\frac{1}{m}\sum_{j=1}^{m}\sqrt{{\left(x_{target}^{i}-x_{gaze}^{j}\right)}^{2}+{\left(y_{target}^{i}-y_{gaze}^{j}\right)}^{2}}$$

### 3.9. Precision

Several Mathematical tools can estimate precision. Among these tools, the root mean square of inter-sample angular distance, also called RMS, is especially useful for this purpose. Precision was calculated using all data samples of both eighteen-point calibration-like and fixation tests by means of the following equation:

(3) $$\theta_{RMS}=\sqrt{\frac{1}{n}\sum_{i=1}^{n}\theta_{i}^{2}}=\sqrt{\frac{\theta_{1}^{2}+\theta_{2}^{2}+\cdots \theta_{n}^{2}}{n}}$$

where $\theta_{i}=\sqrt{{\left(x_{i+1}-x_{i}\right)}^{2}+{\left(y_{i+1}-y_{i}\right)}^{2}}$ represents the visual angle in degrees between successive data samples and *n* is the number of samples in the dataset.

It is important to note that both accuracy and precision were calculated using two data vectors generated by OSCANN. These two vectors are called raw and filtered. This methodology was followed to validate the effect of the filter algorithm implemented with OSCANN. Such an algorithm performs an off-line smoothing by means of a Finite-Impulse Response (FIR) filter with a Gaussian kernel function. More information about data quality and its effects in eye-tracking can be found in [20].

## 4. Results

In previous characterization studies, manufacturers of actual eye-trackers based on VOG technology have reported accuracy values of 0.5${}^{\circ }$ for their devices [24,25]. Providing such information is very important for consumers, mainly because accuracy is considered one of the most important parameters regarding whether or not to choose an eye-tracker. Moreover, the methodologies implemented to carry out those studies have been relevant to perform the study presented in this work. However, it is important to highlight that these accuracy values have often been obtained under ideal conditions, for example, using a chin rest for remote eye-trackers [21]. As a result, during real test conditions, the error of those devices can increase up to one degree more than that reported [15,26]. As mentioned previously, the results presented in this section were obtained using OSCANN under normal environmental conditions to obtain more significant and realistic technical specifications.

The results presented in this section are divided into two sections, namely accuracy and precision. Such a presentation is provided in a top-down manner. That is, an overview of the results from all participants is provided first before presenting relevant results individually. In most cases, unless otherwise indicated, the data presented in each graph represents gaze points estimated using the software of OSCANN. Moreover, all the RMS and absolute errors from all the participants are used for the calculation of μ values.

### 4.1. Accuracy

**Figure 4:**
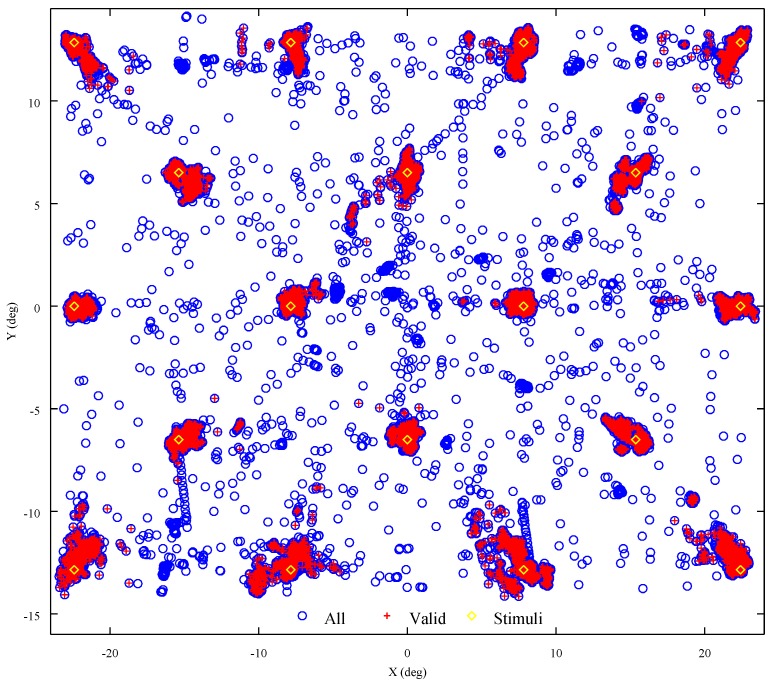
Gaze estimation of twenty-two calibration-like tests of eighteen points.

Figure 4 shows the estimated gaze points from the twenty-two calibration-like tests carried out in this characterization study. Recall that this was the number of volunteers that participated in this study. For better understanding, Figure 4 can be perceived as staying in front of the stimuli monitor. Thus, diamond yellow markers indicate stimuli positions. The first and last stimuli are located at coordinates (−21,11) and (21,−11), respectively. It is worth noting that, in each test, stimuli were shown one at a time in a random order. On the other hand, circled blue markers represent the 58,964 samples of the data set. From this data, points near each stimulus position indicate that the volunteer directed correctly his/her gaze to the corresponding stimulus presented. On the contrary, points away from the stimuli position were excluded since they represent unexpected behavior such as blinks, distractions and anticipations. All these latter points were removed because they can be considered as noise or unexpected behavior. Figure 4 shows both all (blue circles) and valid (plus red markers) gaze positions.

Furthermore, Table 1 shows the standard deviation values of the whole sample set shown in Figure 4. In this table, the lower the mean value, the better the accuracy. Statistical information is divided into two groups to highlight the effect of the internal filter used to smooth the raw data set. As can be observed, such an effect is barely noticeable. This is actually quite remarkable because it means that all relevant information about the recorded gaze position is kept after applying the filter. An overall error of 0.4${}^{\circ }$ was obtained by using OSCANN. These results suggest that OSCANN can be used to estimate gaze position in tasks that demand high accuracy.

**Figure 5:**
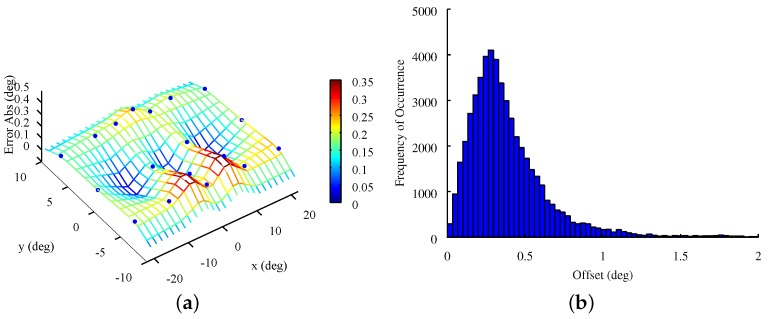
Accuracy distribution and mean value at each stimulus of a calibration-like test of eighteen points. (**a**) Surface plot; (**b**) Histogram.

As stated in the last section, five volunteers were allowed to use contact lenses which makes it possible to analyze the robustness of the pupil detection algorithm of OSCANN. As expected, the incorporation of contact lenses decreased accuracy. The reason is that such lenses add reflections and slightly blur the pupil. Nevertheless, pupil detection was accurate enough for the analysis. In this light, another characteristic that affects accuracy was the stimuli positions. The closer to the border of the screen, the lower the accuracy of the estimation. As was observed, this nature depends on the participant’s physiology. For example, a person with downward pointing eyelashes will generate more occluded eye images when looking toward the borders of the screen. However, if there are no occlusions in such a situation, the impact of stimuli positions on accuracy is close to none. This behavior can be observed in Figure 5a which shows the accuracy information of 52,137 valid samples of twenty-two calibration-like tests. Blue dots, which represent stimuli, are placed on the z axis depending on the error obtained for each position. Points on border regions have considerably higher errors than those near the center. On the other hand, Figure 5b shows the distribution of the accuracy of the same tests. As can be observed, the distribution shows that only a few samples have low accuracy (offset greater than 1 degree).

### 4.2. Precision

This section presents precision data analysis by means of Root Mean Square (RMS). The eye-trackers field of application is determined, among other things, by the precision. For example, to precisely measure fixation and saccadic eye-velocity, an RMS lower than 0.05 is recommended, while for measuring microsaccades the RMS should be 0.03 or lower [23]. On the other hand, the data set used for the formulation of graphs and tables shown in this section was obtained by twenty-two fixation tests. In these tests, volunteers must direct their gaze to a stimulus located at the center of the stimuli monitor. Such a data set comprises 33,710 data samples.

**Figure 6:**
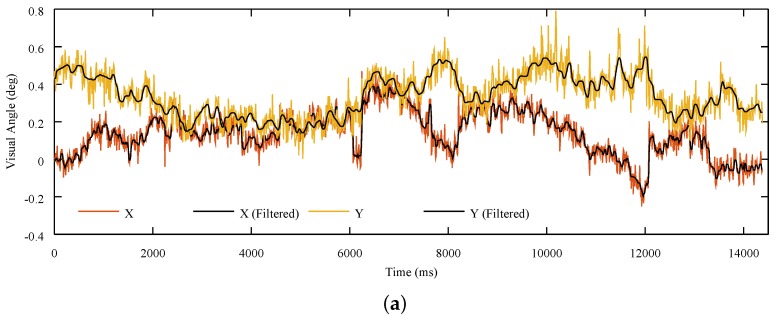

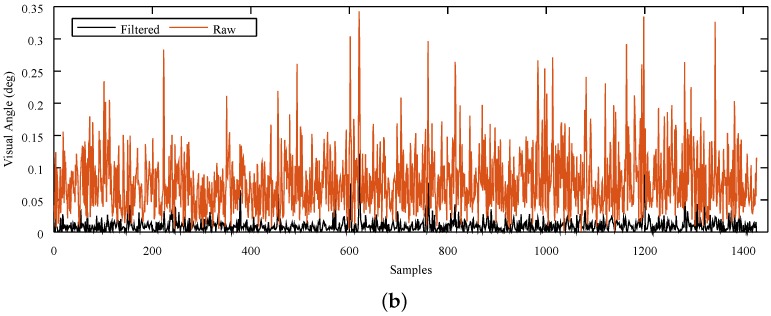
Angular position and distance estimation of the data samples of one volunteer. (**a**) Gaze Position; (**b**) Angular Distance.

Figure 6a shows the angular positions estimated from the data set of one fixation test. As can be observed, OSCANN provides angular information of both components x and y. The samples shown in this figure represent the behavior of one volunteer. In this particular test, there were neither blinks nor unexpected movements. This claim was corroborated by means of the stored video. In addition, Figure 6b depicts angular distance between consecutive samples. This graph shows the high precision obtained with OSCANN after filtering the data. This is because the filter is able to reduce the RMS from around 0.10 to 0.03.

Table 2 summarizes statistically all of the data samples. As previously mentioned, this information is divided into all and valid data samples. In these results, the higher the value of μ, the lower the precision. Results show that OSCANN is a highly precise sensor with a precision slightly higher than 0.03${}^{\circ }$ RMS. Note that the precision is better using valid data samples than all data samples. This is because the filter removes a great deal of noise of the raw data.

Statistical values in Table 2 only represent the precision of the middle point of the stimuli monitor. In this position, eye occlusions are scarcely ever produced. Moreover, the shape and size of the pupil are appropriated for the purpose of detection. Therefore, in order to provide a much more realistic precision value of OSCANN, the calibration-like tests of eighteen points were used.

**Figure 7:**
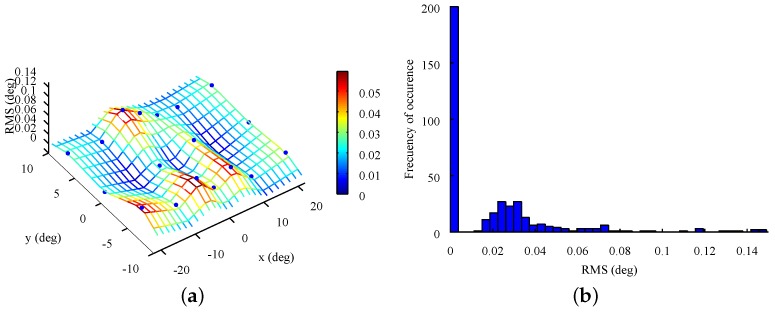
Mean RMS for each target from the calibration-like tests. (**a**) Surface plot; (**b**) Histogram.

Figure 7 plots 396 RMS values obtained from the 52,137 samples of the twenty-two calibration-like tests of eighteen points. Thus, the surface depicted in Figure 7c shows the mean precision at each point of these tests. As can be observed, precision is affected at the right edge of the stimuli monitor. This behavior was expected because directing the gaze to that part of the monitor usually results in eye occlusions. Even so, the precision obtained on the whole stimuli surface was excellent. On the other hand, as can be seen in the distribution presented in Figure 7d, it is centered below 0.03${}^{\circ }$. This fact demonstrates the good quality of the results. Finally, Table 3 shows the statistical data on the precision of the twenty-two calibration-like tests of eighteen points. Comparing its values to those in Table 2, it can be concluded that the precision decreases when the entire screen is used. It is worth mentioning that the mean value of 0.048${}^{\circ }$ RMS is still under the recommendation of 0.05 for high precision eye-trackers. Thus, if the study were to look for small involuntary movements, it is better to present only the stimuli near the center of the monitor.

Finally, a fixation test of thirty seconds was performed using the artificial eye described in Section 3.4. The precision obtained in this experiment was of 0.0033${}^{\circ }$ and 0.0604${}^{\circ }$ RMS for the valid and all data samples, respectively. These values confirm the high precision of OSCANN and prove the low level of noise of the camera utilized.

## 5. Conclusions

This work describes a complete regulated solution for clinical practice named OSCANN. This solution aims at providing precise analysis and objective evaluations of eye movements. To this end, it is comprised of several hardware components and software modules. In terms of hardware, it includes an eye-tracker sensor based on non-invasive VOG technology coupled with other components to capture pure oculomotor movements. Regarding its software, modules are included for controlling, visualizing and managing neurological oculomotor examinations. It is worth noting that this medical solution mitigates two major disadvantages of the actual commercial eye-tracker: cost and usability. In addition, its simple and friendly user interface enables it to be used by everyone, whether a hardware and software novice or expert.

On the other hand, prior work has characterized commercial eye-trackers in terms of accuracy and precision. For example, in [21], the technical characterization of an eye-tracker was reported. These types of studies of actual commercial eye-trackers report values of 0.5${}^{\circ }$ and 0.005${}^{\circ }$ for accuracy and precision, respectively.It is important to mention that the methodologies implemented in those characterization studies were carried out under ideal environmental conditions. Such situations include the use of extra components for head fixation. This work describes the methodology utilized to perform the characterization study of OSCANN. Such a study reported values lower than 0.4${}^{\circ }$ and 0.035${}^{\circ }$ of accuracy and precision, respectively. Additionally, OSCANN does not need any extra component for tracking the eye; it is an all-in-one device prepared for the clinical environment.

Finally, it is important to highlight that OSCANN captures 100 frames per second. This rate is appropriate for the application field in which this solution is intended to be used. However, future work should include an approach to increase this rate to more than 320 frames per seconds. This frame rate is needed to capture some special features of eye movements such as microsaccades. This increase in velocity is challenging due to the quantity of information that must be captured, displayed and stored at the same time. One possibility to resolve this is the implementation of a parallel or distributed software solution. 

## Figures and Tables

**Table 1:** Statistical results of the accuracy using the data samples of the calibration-like tests of eighteen points. The number of volunteers is indicated in parenthesis; floating-point values represent angular distances in degrees.

Data Set											
Volunteers	A					Volunteers	B				
	Valid			All			Valid			All	
	μ	σ		μ	σ		μ	σ		μ	σ
Normal (8)	0.314	0.188		0.325	0.190	Normal (9)	0.427	0.384		0.457	0.493
Lens (3)	0.565	0.611		0.621	0.753	Lens (2)	0.543	0.538		0.622	0.900
All (11)	0.383	0.376		0.406	0.447	All (11)	0.445	0.404		0.483	0.581

**Table 2:** Statistical results of the precision using the data samples of the fixation tests. The number of volunteers is indicated in parenthesis; floating-point values represent angular distances in degrees.

Data Set											
Volunteers	A					Volunteers	B				
	Valid			All			Valid			All	
	μ	σ		μ	σ		μ	σ		μ	σ
Normal (8)	0.034	0.011		0.130	0.019	Normal (9)	0.039	0.023		0.236	0.247
Lens (3)	0.027	0.024		0.101	0.013	Lens (2)	0.040	0.006		0.114	0.012
All (11)	0.032	0.014		0.122	0.021	All (11)	0.036	0.016		0.151	0.081

**Table 3:** Statistical results of the precision using the data samples of the calibration-like tests of eighteen points. The number of volunteers is indicated in parenthesis; floating-point values represent angular distances in degrees.

Data Set											
Volunteers	A					Volunteers	B				
	Valid			All			Valid			All	
	μ	σ		μ	σ		μ	σ		μ	σ
Normal (8)	0.038	0.049		0.171	0.408	Normal (9)	0.053	0.039		0.467	0.795
Lens (3)	0.075	0.104		0.443	0.717	Lens (2)	0.153	0.189		0.962	1.204
All (11)	0.048	0.069		0.244	0.521	All (11)	0.071	0.079		0.557	0.668
